# From Molecular to Multiasperity Contacts: How Roughness Bridges the Friction Scale Gap

**DOI:** 10.1021/acsnano.2c08435

**Published:** 2023-01-23

**Authors:** Lucas Frérot, Alexia Crespo, Jaafar A. El-Awady, Mark O. Robbins, Juliette Cayer-Barrioz, Denis Mazuyer

**Affiliations:** † Department of Physics and Astronomy, Johns Hopkins University, 3400 N. Charles Street, Baltimore, Maryland21218, United States; ‡ Department of Mechanical Engineering, Johns Hopkins University, 3400 N. Charles Street, Baltimore, Maryland21218, United States; § Laboratoire de Tribologie et Dynamique des Systèmes, École Centrale de Lyon, CNRS UMR5513, 69134Ecully, France

**Keywords:** friction, transient, response, roughness, contact junction, fatty acid monolayers

## Abstract

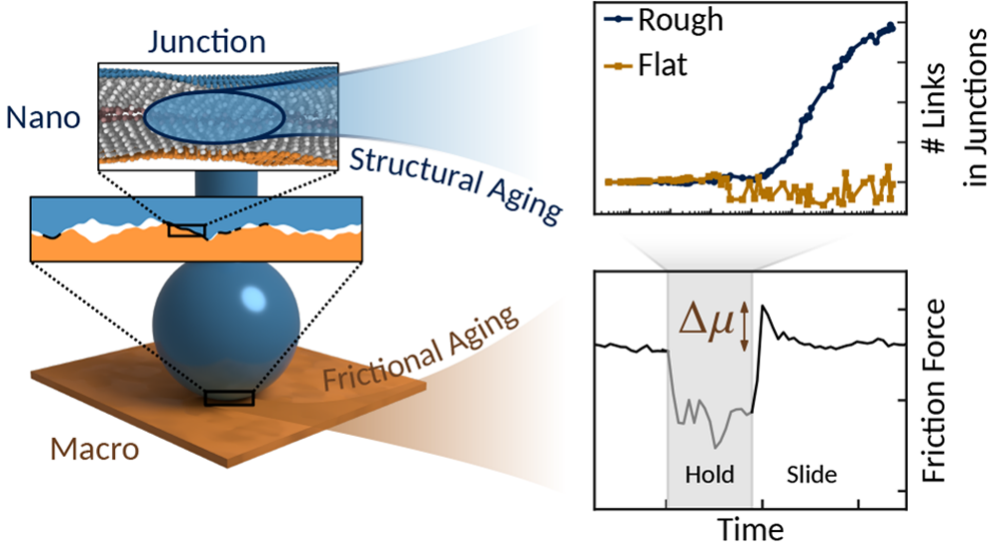

The tangential force required to observe slip across a whole frictional interface can increase over time under a constant load, due to any combination of creep, chemical, or structural changes of the interface. In macroscopic rate-and-state models, these frictional aging processes are lumped into an ad hoc state variable. Here we explain, for a frictional system exclusively undergoing structural aging, how the macroscopic friction response emerges from the interplay between the surface roughness and the molecular motion within adsorbed monolayers. The existence of contact junctions and their friction dynamics are studied through coupled experimental and computational approaches. The former provides detailed measurements of how the friction force decays, after the stiction peak, to a steady-state value over a few nanometers of sliding distance, while the latter demonstrates how this memory distance is related to the evolution of the number of cross-surface attractive physical links, within contact junctions, between the molecules adsorbed on the rough surfaces. We also show that roughness is a sufficient condition for the appearance of structural aging. Using a unified model for friction between rough adsorbed monolayers, we show how contact junctions are a key component in structural aging and how the infrajunction molecular motion can control the macroscopic response.

Friction is a phenomenon that affects the behavior of virtually every mechanical system: from the movement of geological faults that can cause earthquakes to the sliding of atomic force microscopy tips. In these systems, friction is intimately linked to contacting asperities:^1,2^ the inevitable roughness of natural and manufactured surfaces implies that the true contact interface is made up of a sparse set of contact junctions^3^ which govern the frictional response,^2^ as well as other tribological phenomena.^4−6^

At macroscopic scales, the static friction force has been observed to increase logarithmically with resting contact time for amorphous materials, including woods,^7^ rocks,^8^ and polymers.^3^ In general, this can attributed to any combination of the following effects: an increase of the true contact area due to a mechanical creeping of contact spots^3^ (geometrical aging), a change in interaction energy between the surfaces^9^ via chemical changes^10,11^ (chemical aging), and structural/physical changes^12^ (structural aging). Upon sliding, the contact interface rejuvenates over a characteristic sliding distance *D*_0_.^13,14^ Such behavior is widely modeled using rate-and-state friction,^8,15,16^ which describes the friction force in terms of a state variable ϕ, which represents the average age of the microcontacts and whose evolution equation encompasses aging and rejuvenation. Despite recent efforts to relate rate-and-state parameters to the physics of rough surfaces,^11,17−20^ in practice *D*_0_ remains a phenomenological variable fitted to laboratory experiments.

Geometrical aging, due to creep, has been extensively discussed in its effects on the macroscopic friction response,^8,15^ and both chemical and structural (or physical) agings have been shown to increase the “contact quality”: i.e., the junction shear strength.^9,10,12^ To our knowledge, no attempt has been made to explain the latter’s underlying molecular mechanisms *and* how they interact with surface roughness to produce structural aging and rejuvenating.

Our aim here is therefore 2-fold: elucidating the influence of roughness on these nanoscale friction mechanisms and integrating the physical contribution of these mechanisms into a macroscopic friction description. We focus on a model system representative of structural aging: two rough cobalt surfaces coated with a stearic acid (C_17_H_35_COOH, commonly used as an environmentally friendly lubricant) in dodecane (C_12_H_26_) dilute solution. After deposition of the solution, the stearic acid adsorbs on the surfaces and forms a monolayer.^21,22^ These two rough monolayer-covered surfaces are brought into contact in our molecular tribometer^23^ at a constant normal force. A slide–hold–slide protocol is applied with constant velocity and varied hold times. Molecular dynamics (MD) simulations reproducing the experimental protocol (at shorter time scales) are used to probe the details of the contact interface, for which we combine the two surfaces roughness profiles into a single rough-on-flat setting (roughness profiles are generated using measurements of the experimentally used surfaces). Nanoscale mechanisms uncovered with MD and experimental results are used to establish a unifying friction model that we show reproduces the transient friction behavior observed in experiments.

## Results

**Figure 1:**
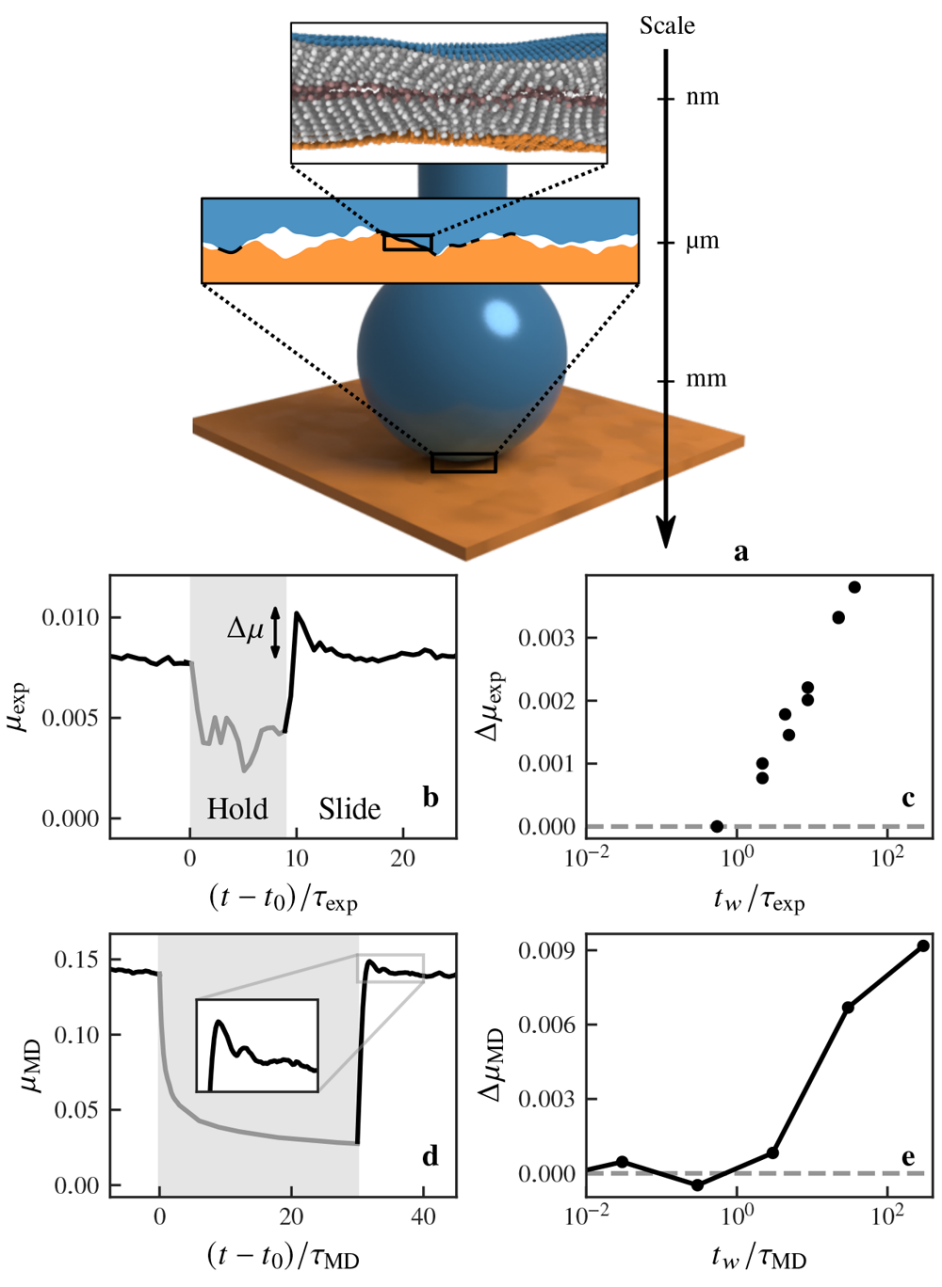
Transient friction behavior of stearic acid monolayers. (a) Schematic of a ball-on-flat contact experiment for fatty-acid monolayers showing the multiscale nature of friction. The apparent contact area has a radius of 3.52 μm, while the true contact area is made up of sparse junctions where the adsorbed monolayers interact due to the surface roughness. Friction response of (b, c) the experiments (±10% error) and (d, e) simulations, respectively. (b) and (d) show the transient friction behavior in a slide–hold–slide protocol, with hold highlighted in gray (*t*_0_ being the start time of the hold stage). An overshoot of the steady-state friction force can be observed at the onset of the second slide stage. (c) and (e) show that the magnitude of the overshoot increases with the hold time, *t*_w_, if it is greater than a relaxation time of τ_exp_ = 2.2 s in the experiment and τ_MD_ = 0.8 ns in the simulations.

Figure 1a illustrates the multiscale aspect of friction of surfaces coated with fatty acid monolayers: the inevitable roughness of the surfaces in contact partitions the apparent contact interface into contact junctions,^3^ where the fatty acid molecules are close enough to interact. This occurs, as we show in this work, even with a root-mean-square (RMS) roughness as small as 0.6 nm, as measured in the current experiments with atomic force microscopy (AFM) over 1 μm^2^. Figure 1b,d shows the transient friction response of stearic acid monolayers for a slide–hold–slide (SHS) protocol, where *t*_0_ is the start time of the holding stage and μ_exp_ and μ_MD_ are the ratios of tangential to normal force for the experiment and simulation, respectively. During the holding stage, relaxation occurs and the tangential force decreases to a nonzero value.^9^ After rest, when the sliding resumes at the velocity prior to the hold phase, the friction force overshoots the steady-state value by Δμ_exp_ (respectively Δμ_MD_). This overshoot is observed in both the experiments and MD simulations of rough-on-flat (cf.Figure 1d) and rough-on-rough (cf. Figure S1 in the Supporting Information) and is consistent with previous observations of frictional aging in experiments^7,8,12,13^ and simulations^10^ at a macroscopic scale. Figure 1c,d shows that for both the experiments and the simulations the magnitude of the overshoot increases with the waiting time, *t*_w_, for times longer than the relaxation times τ_exp_ = 2.2 s and τ_MD_ = 0.8 ns of the experiment and simulation, respectively. We have defined τ_exp_ directly from Figure 1c, but τ_MD_ is defined from scaling regime changes in the mean-square displacement of monomers in an equilibrium simulation (see Figure S2), hence our interpretation of τ_exp_ and τ_MD_ as relaxation time scales. An independent simultaneous measurement of the tangential stiffness in the SHS experiment^24^ shows a reversible increase of the stiffness during the hold step (see Figure S3). This, combined with previous observations of the decreasing film thickness at rest,^14^ confirms the presence of structural aging at the molecular scale during rest. With both our experimental and computational systems showing evidence of aging, we investigate the role of roughness in the observed transient friction response.

**Figure 2:**
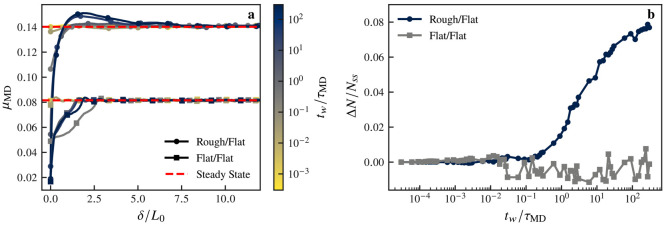
Effects of roughness on the transient friction response. (a) Comparison of the transient friction response of rough-on-flat and flat-on-flat systems after a hold time *t*_w_ (darker curves have longer *t*_w_). The flat/flat system shows that the friction force recovers a steady-state value without overshooting, unlike the rough/flat system, which exhibits a friction force peak above μ_ss_ for large *t*_w_. (b) Comparison of the increase in the number of cross-surface links (Δ*N*) in the holding stage. While Δ*N* increases markedly for the rough/flat system, which indicates structural aging, it remains constant in the flat/flat system.

We compare in Figure 2a the MD transient friction response (as a function of sliding distance, δ, normalized by the molecular length, *L*_0_ = 2.14 nm) of the rough-on-flat system shown in Figure 1 to a system with identical monolayers on two atomically flat surfaces. Unlike the rough-on-flat system, this flat-on-flat system does not overshoot the steady-state friction level, regardless of waiting time *t*_w_, indicating that the system does not age. To quantify the aging difference between the flat and rough systems, we plot in Figure 2b the total number, *N*, of attractive interactions that atoms of one surface have with the other. We call these interactions cross-surface links: they are van der Waals bonds between molecules belonging to different surfaces. The relative change in *N*, compared to the steady-state value *N*_ss_, while the system is at rest gives a metric for the aging process: it is apparent from Figure 2b that in the flat system *N* stays constant (with thermal noise) while the rough system sees its number of cross-surface links increase over the resting period (300 τ_MD_). While an increase, e.g. due to creep, of the true contact area could cause this, we show in Figure S4 that the footprint of the contact does not evolve during rest in our simulations, and the increase in tangential stiffness measured in experiments (Figure S3), in conjunction with the relative compliance of the monolayers compared to the substrate, also excludes this mechanism in our empirical observations. We are therefore quantifying the structural age of the system. The difference between the rough and flat systems can be explained by the dynamics of the contact junctions that occur in the rough system: at the edges of these junctions the surfaces are close enough that molecules in the vicinity of the contact can have their tail move in and out of the contact, thereby providing degrees of freedom for the system to evolve. This does not occur in the flat case, where all molecules are already participating in the contact. The similarity between the rough-on-flat curve in Figures 2 and 1e seems to reinforce the relationship between age, friction, and cross-surface links, analogously to how entanglement density controls the interface strength in polymer welding.^25^ We now investigate this relationship in the sliding phase to understand how the interface rejuvenates.

**Figure 3:**
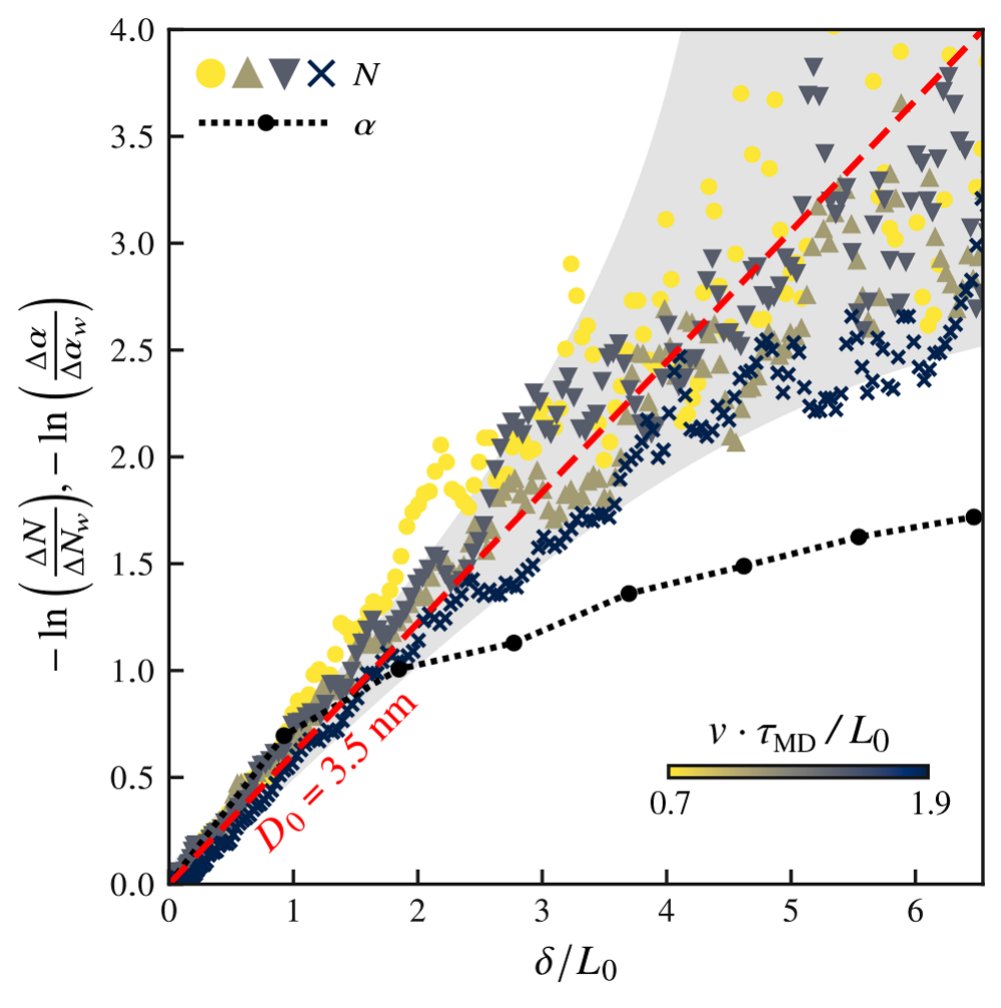
Evolution to steady state of the number of cross-surface links (*N*, symbols), and contact survival fraction (α, dashed line with circles) in slide-after-hold as a function of the sliding distance δ. The dashed (red) line shows a decay to steady state of the form exp(−δ/*D*_0_) with *D*_0_ = 3.5 nm. This decay holds well for *N* even for several times *D*_0_, regardless of sliding velocity (symbol shapes, *v*τ_MD_/*L*_0_ = 0.7, 1.2, 1.4, 1.9, from light to dark shades, all with *t*_*w*_/τ_MD_ = 300), suggesting that *N* is a representative quantity of the interface state, since the experimental decay distance to steady state is 4.8 ± 1.4 nm and the memory function based on the renewal of the microcontact geometry, α, decays more slowly. Dispersion of the data on long sliding distances is expected due to the natural noise of the systems, which introduces uncertainty in the steady-state estimate (gray area).

We show in Figure 3 how the number of cross-surface links *N* returns to its steady-state value as a function of the sliding distance δ. Symbols show *N* for different sliding velocities (*v*τ_MD_/*L*_0_ = 0.7, 1.2, 1.4, 1.9, from light to dark shades). We compare *N* to another memory function, the survival contact fraction α. It defines, as a function of sliding distance, how much of the contact interface between two rough surfaces is common to the interface when the system was at rest: i.e., α = |*A*(δ)∩*A*(0)|/|*A*(0)| with *A*(δ) being the set of contact points at a given sliding distance. This memory function postulates that the rejuvenation of the contact comes from the geometric renewal of the microcontact population.^3^ We define Δ*N* = *N*(δ) – *N*_*ss*_ (respectively Δ*α* = α(δ) – α_ss_) and Δ*N*_w_ = *N*(0) – *N*_ss_ (*idem* for Δ*α*_w_). The quantity –ln(*Δ*N/Δ*N*_w_) gives a measure of the rate at which the system rejuvenates: rate-and-state models that use the aging law ϕ = 1 – *v*ϕ/*D*_0_ predict that ϕ(δ) – ϕ_*ss*_ ∝ exp(−δ/*D*_0_) . In Figure 3, axes are chosen so that an exponential decay is a straight line with slope 1/*D*_0_. We show that α, which represents a memory definition based on the geometry of the contact, decays to a steady state at a much lower rate than the number of cross-surface links. The latter decays in good agreement with an exponential decay having *D*_0_ = 3.5 nm. This is consistent, in magnitude, with the distance needed for the experimental friction force to return to steady state, measured to be 4.8 ± 1.4 nm. Although the number of cross-surface links and the friction force should not be directly compared, the MD simulations still reproduce a value of *D*_0_ independent of sliding velocity and in the same order of magnitude as the experiment, despite the 10 orders of magnitude difference in sliding velocity between simulations and experiments. Furthermore, no simulation parameter was adjusted to match the experimental data.

Uncertainty (due to noise) in the measurement of the steady-state value of an exponential decay causes deviations from the straight line. The gray area in Figure 3 shows the deviation extent based on the noise in *N* measured in the simulations. The rejuvenation difference between *N* and α indicates that *D*_0_ is not an intrinsic property of the junction sizes, as is commonly interpreted,^3^ but rather a velocity-independent system property^11,20^ that combines the surface roughness and the molecular organization of the fatty-acid molecules. Note that in systems where two or more aging mechanisms contribute to the transient friction response, *D*_0_ may be the result of more complex interactions that are absent from our experiments.

**Figure 4:**
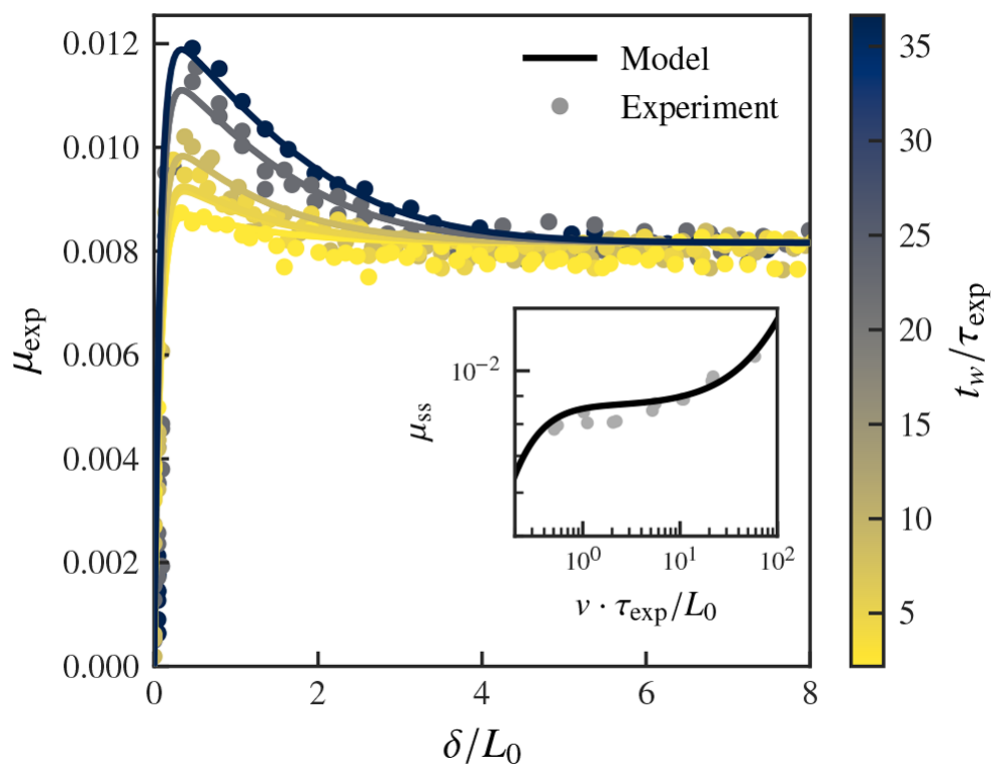
Transient friction derived from a multiscale friction model. The theoretical response (black line) is compared to the experimental friction transient (*v* = 0.5 nm/s) and steady-state (inset) responses (circles). The characteristic times of the nanoscale contribution to the friction force are consistent with measured relaxation times (cf. Figure 1), and the model is capable of reproducing the transient friction behavior as well as the stationary response.

Our experiments and simulations show that taking the surface roughness into account, even at the nano scale, and by extension the formation of contact junctions, is a key component in structural frictional aging, although junctions could arise from other sources of heterogeneity, such as imperfect surface coverage of the adsorbed layer.^9^ We also demonstrate that the cross-surface link formation and destruction within contact junctions govern key aspects of the aging process and of the transient frictional response. We combine these two ideas into a model that links the nanoscopic and macroscopic scales and analytically reproduces the steady-state friction response as well as the transient overshoot in the presence of roughness. This model unifies existing approaches at two length scales: the macro scale where the true contact area is made up of monolayer junctions due to the presence of surface roughness and the scale of molecular interactions within a junction. At the molecular scale, we use the theory developed in ref (26) for adhesive friction of polymer chains. This study postulated that chains at the interface are in either a bound state or free state and that the transition from bound to free can occur by thermal fluctuations or an external force. Three characteristic times govern the state transitions: the time to break a molecular link (i.e., cross-surface link), the time to (re)activate a molecular link, and the delay time related to the withdrawal of a link from the contact zone.^27^ Chernyak and Leonov assumed a stationary stochastic process and constant surface separation to compute steady-state values of the shear stress within a contact junction, as a function of sliding velocity, σ_ss_(*v*). At the macro scale, we define the interface age ϕ, which follows the state law mentioned above, and the aging factor^28^*f*_a_(ϕ) = 1 + ω ln(1 + ϕ/τ_1_) . The contact is made up of contact junctions totaling a true contact area *A*_r_. This is sufficient for a calculation of the macroscopic steady-state friction force *F*_t,ss_. In the inset of Figure 4, we show our fit of *F*_t,ss_ to the steady-state experimental values of the friction force at different sliding velocities (values of the model parameters, both measured and fitted, are given in Methods). For the characteristic detachment time, we find values in the same order of magnitude as the relaxation time measured in Figure 1, and as values measured for different organic monolayers with the same thickness.^12^ To account for transient effects at the onset of sliding, i.e. on sliding distances shorter than *D*_0_, the elastic tangential response of the asperities in contact is approximated with Mindlin’s theory of elastic spheres in frictional contact,^29,30^ extended in ref (31) to a Greenwood–Williamson approach,^5^ which approximates the junction distribution. The resulting friction force is expressed as *F*_t_(*v*,*t*) = *f*_a_(ϕ(*v*, *t*))(1 – exp(− *vt*/δ*))*A*_r_σ_ss_(*v*), where the exponential term models the transition from elastic tangential response (stick) to the slip regime and δ* is the ratio of the steady-state friction force to the measured tangential stiffness of the interface. The hypotheses leading to the full derivation of this equation are given in Methods. These ingredients provide a good fit to the experimental data in the stationary and transient regimes, by accounting for the time, sliding velocity, surface roughness, and elastic properties of the monolayers, as illustrated in Figure 4. Without introducing contact junctions, the proposed approach predicts no overshoot of the stationary friction value, in agreement with our MD simulations. This demonstrates that the physics of friction between the monolayers is well captured by the coupling of link formation inside contact junctions, governed by the aforementioned characteristic times, and the sliding dynamics of the junctions themselves all over the contact area. Thus, the interface accommodates the shearing through a combined effect of roughness and molecular interactions. This approach can readily be generalized to other systems with adsorbed organic layers and rough surfaces, which are commonplace in microelectromechanical systems^12^ and biomechanics:^32^ e.g., for natural or artificial joints where proteins can form a protective layer on a hard substrate.^33^

## Conclusions

Combining experiments and simulations of friction between fatty-acid monolayers deposited on rough surfaces, complemented by a multiscale theoretical approach, we were able to probe the molecular mechanisms underlying structural frictional aging and its transient friction response and bridge the scale gap to the macroscopic friction behavior. We have shown, for monolayers adsorbed on rough, stiff surfaces, that the stiction peak and its decay are controlled by molecular mechanisms within contact junctions, not the sizes of the junction themselves. To uncover these molecular mechanisms, we have demonstrated that in the absence of contact junctions (e.g., due to the absence of roughness), structural aging disappears. The system aging can then be explained by the capacity of molecule tails to come in and out of contact junctions, which cannot happen when surfaces are flat at the atomic scale. This sheds light on the memory length scale *D*_0_ with important implications on a broad range of frictional systems. Our findings highlight the importance of both surface roughness at the molecular scale and molecular mechanisms at the macroscopic scale. We combined these aspects into a multiscale theoretical model that correctly reproduces the transient friction overshoots observed in experiments and whose principles are generalizable to a wide variety of frictional systems with surface roughness and coatings, such as biomechanical systems, e.g. cartilaginous or artificial joints, for which roughness can dramatically alter the proper function and lifetime.^34^

## Methods

### Experimental Friction Measurement

Using stearic acid (99.0% purity, from Sigma-Aldrich) with dehydrated and filtered dodecane, a dilute solution was prepared at a concentration of 0.002 mol/L. The surfaces consisted of a fused silicate glass sphere of radius 2.030 ± 0.005 mm and a ⟨100⟩ silicon wafer. The latter was cleaned with isopropanol and deionized water using a spin-coater at 8000 rpm and then dried under a nitrogen flow. Both surfaces were then coated with a 40 nm thin cobalt layer by means of a cathodic sputtering system under low argon pressure (10^–6^ mbar). Experiments were conducted by sliding the sphere over the plane using an ATLAS molecular tribometer.^23^ A typical friction experiment was performed by approaching the sphere toward the plane, confining the stearic acid monolayers on each surface until a constant normal force of 0.70 ± 0.01 mN: i.e., a corresponding maximum Hertzian contact pressure of 27 MPa (at a normal velocity of 0.1 nm/s). Then without breaking contact, a slide–hold–slide procedure was used: the sphere slid over the plane over a few hundreds of nanometers at a constant sliding velocity of 0.5 nm/s and then was held stationary for a time, *t*_w_, before resuming the lateral displacement. Hold times were varied between 1 and 120 s. During the experiment, the response to a superimposed oscillating sphere displacement in both directions, normal and tangential, of amplitude 0.1 nm and 38 Hz (respectively 0.03 nm and 70 Hz) provided, without disturbing the friction process, the stiffness and the viscous damping of the confined interface in both directions.^23^ All measurements were carried out in a sealed chamber with a relative humidity lower than 1% and *T* = 23.0 ± 0.5 °C under an argon atmosphere.

### Surface Topography Characterization

Multiscale characterization of the surface topography was performed before and after the experiment to ensure no surface damage. AFM measurements of the surface topography over an area of 1 μm × 1 μm provided an RMS of surface heights of 0.6 nm and a radially averaged power-spectrum density (PSD) as shown in Figure S6. At a larger scale, a Bruker interferometry profilometer provided an RMS value of 0.5 nm on both surfaces in Phase Shift Interferometry mode over an area of 63 μm × 47 μm.

To generate synthetic rough surfaces from the measured surface profile, we used the PSD computed from the AFM data. We cut off long-wavelength modes as necessary to generate a smaller surface, i.e. for MD simulations that were 200 nm × 200 nm, and used uniformly distributed phases^35^ to produce surfaces with the same (or reduced) spectral content as the surface used in experiments. Full-size (1 μm × 1 μm) surfaces were used for continuum simulations of dry elastic contact, while a reduced size (200 nm × 200 nm) was used for MD simulations.

### Molecular Dynamics

Molecular dynamics simulations were conducted using coarse-grained potentials^36^ adjusted for alkane chains with one bead corresponding to two CH_2_ groups. Stearic acid chains consisted of nine beads. Head groups were positioned on a hexagonal lattice with spacing 5.5 Å. The top lattice was rotated 90° to avoid commensurate effects in the flat/flat friction response. The applied normal pressure was *p̅* = 27 MPa. Roughness was applied to the head group lattice by vertical displacement of the beads and their connected chain. The system was initially equilibrated at *T* = 300 K, with the surfaces separated, using a Langevin thermostat and a time step of Δ*t* = 1 fs. Surfaces were brought together with the applied normal pressure and equilibrated again. Sliding of the top head group lattice was done via a spring attached to its center of mass. The stiffness of the spring was such that the period of the mass-spring system was 3.5 ps. In the initial sliding phase, the free end of the spring slid at velocity *v* for 600 Å and Δ*t* = 1.25 fs. The system was then allowed to rest by setting *v* to zero for 30 ns with Δ*t* = 3 fs. Restart of the sliding was done by setting *v* back to its original value with Δ*t* = 1.25 fs. The friction force was measured as the force in the spring, and the temperature was controlled at 300 K with a Langevin thermostat acting on the degrees of freedom normal to the sliding direction. The number of cross-surface links was computed with a radius cutoff of 10 Å for attractive links and 5 Å for compressive links, which corresponded to the potential cutoff and equilibrium distance, respectively. All simulations were conducted with the open-source software LAMMPS.^37,38^ Figures were generated with Blender, Ovito,^39^ Matplotlib,^40^ Scipy^41^ and Numpy.^42^ The source code of simulations and figures is available.^43^

### Continuum Elastic Rough Contact

A Fourier-based boundary integral approach^44,45^ was used with a projected conjugate gradient algorithm^46^ to solve the elastic rough contact problem. The linear elastic material properties used were determined from the experiments:^21^ the contact Young’s modulus E*= *E*/(1 – ν^2^) was set to 48 GPa and the average pressure was set to *p̅* = 27 MPa. The contact problem was solved with a compound roughness^30^*h* = *h*_2_ – *h*_1_ from two generated surfaces *h*_1_ and *h*_2_, the latter of which was shifted by δ, the sliding distance. The true contact area was the area where contact pressure was strictly positive. The survival fraction at δ was the normalized magnitude of the area in common with the initial contact area. All simulations were conducted with the open-source library Tamaas.^47,48^

### Junction-Based Friction Model

The friction force per junction in our model was expressed as the product of a velocity and age-dependent shear stress σ, by the junction area *A*_*j*_: *F*_*j*_(*v*,*t*) = σ(*v*,ϕ(*t*))*A*_*j*_, where ϕ is the junction age typically defined in rate-and-state models^15,28^ and obeys the state equation ϕ = 1 – *v*ϕ/*D*_0_. Its contribution to the shear stress comes in the form of an aging factor, i.e. σ(*v*,ϕ) = *f*_a_(ϕ)σ_ss_(*v*), with *f*_a_(ϕ) = 1 + ω ln(1 + ϕ/τ_1_) from ref (28) and σ_ss_ given in ref (26). This decomposition of σ is due to Chernyak and Leonov’s assumption of a stationary stochastic process for the attachment and detachment of molecules at the interface to compute the velocity-dependent shear stress, which excludes aging.

The macroscopic friction force *F*_t_ is given by the sum of *F*_*j*_ over all contact junctions. For the sake of simplicity, we assumed all junctions to have the same age ϕ. We approximated, for each junction, the transition from stick to slip with the model derived by Mindlin^29,30^ for the frictional contact of elastic spheres. The application of Mindlin’s model^5^ to a multiasperity approach proposed in ref (31) was used here, but more accurate models for rough surface contact, such as boundary integral simulations, could be employed.

To summarize the provenance of each contribution to the macroscopic tangential force:

- The velocity-dependent steady-state shear stress was computed using the friction model devised in refs (26 and 27) based on the dynamics of molecular links breaking/formation during sliding
- Rate-and-state models^8,15,18,28^ gave the aging contribution.
- The elastic response of asperities for short tangential displacements was approximated with a spherical frictional contact model developed in ref (29) and extended in ref (31) to a ref (5) approach.

We now go through the procedure we used to determine each parameter of the model.

#### In the Steady-State Regime

The Chernyak–Leonov theory is used to describe the friction between the stearic acid molecules, according to three elementary times:^27^ τ_0_, the time necessary to break a link, τ, the time necessary to form a link, and τ̂, the time for a molecule to withdraw from the interpenetration zone. According to this model, the interfacial shear strength σ_ss_ can be written as
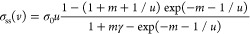
with *u* = tan χ*vτ*_0_/(2*L*_0_), *m* = τ/τ_0_, γ = τ/τ̂, and σ_0_ = (2G/tan χ)(*L*_0_/*L*_H_) deduced from ref (27), where χ is the angle made by the stretched molecule in sliding. In the expression of σ_ss_, only τ_0_, γ, and χ are free parameters that require fitting: *m* can be computed using Figure S2, while *G* and *L*_H_ are measured^23^ and *L*_0_ can be found in the literature.^49^ The coefficients ω and τ_1_ in *f*_a_ can be independently fitted from Figure 1, and the values are given in Table 1. Assuming a an average pressure within contacts of 700 MPa, lower than the cobalt hardness, we find that both τ_1_ and τ_0_ have values in the same order of magnitude as τ_exp_ = 2.2 s and values reported in the literature for different organic compounds but similar monolayer thicknesses,^12^ confirming that they relate to a chain relaxation mechanism as postulated in ref (26). We also find that γ is effectively zero, indicating that the retraction time τ̂ is very large compared to the attachment time τ.

**Table 1:** Numerical Values Used for the Junction-Based Friction Model

measd value	steady-state param	aging param
*L*_0_ = 2.14 nm	τ_0_ = 5.37 ± 0.89 s	τ_1_ = 5 s
*L*_H_ = 1.55 ± 0.2 nm	γ = 0.0	ω = 0.278
*G* = 6.3 ± 1.2 MPa	χ = 55.3 ± 1.6°	
δ* = 0.16 nm		
*D*_ss_ = 4.8 ± 1.4 nm		
*m* = 2.5 × 10^–3^		
*K*_*x*_ = 43 kN m^–1^		

#### Onset of Sliding

The macroscopic transient friction force for an interface transitioning from rest to a sliding velocity *v* is calculated from the extension of Mindlin’s theory to rough surfaces.^31^ This is possible because the transient friction behavior is observed over sliding distances much smaller than the characteristic diameter of the contact junctions (see Figure S5). In a multiasperities interface, this means some contact spots remain in partial sliding while others are moved in total sliding. For a single spherical junction, Mindlin determined that the elementary tangential force *f* required to move a microcontact in partial sliding is simply *f* = *μ f*_n_[1 – (1−δ/δ*)^3/2^], where *f*_n_ is the normal load applied on one microcontact and δ* is the applied tangential displacement necessary for full sliding. We can obtain it from our *in situ* tangential stiffness measurements:^50^ δ* = *F*_t,ss_/*K*_*x*_. The Greenwood–Williamson model applied to the multicontact interface^31^ gives the global force contribution *F*_t_ = *A*_r_(1–exp(−*vt*/δ*))*f*_a_(ϕ(t))σ_ss_(*v*) .

The remaining model parameter is *D*_0_, which we can estimate from experiments. The measured distance, denoted *D*_ss_, required for the friction force to return to its steady-state value can be used to identify the distance necessary in the model for the force to be within 90% of its steady-state value, thereby estimating *D*_0_. This threshold corresponds to the error in the steady-state response of the experiment.

## Supporting Information Available

Presence of structural aging and exclusion of other aging modes and additional data on surface roughness, relaxation time scales, and memory length scale (PDF)
