# Supplementary material for: From Molecular to Multiasperity Contacts: How Roughness Bridges the Friction Scale Gap
Source: ACS Nano. 2023 Jan 23;17(3):2205–11. doi: 10.1021/acsnano.2c08435 (PMC9933612; doi:10.1021/acsnano.2c08435)
Supplement: Supplementary file 1 — nn2c08435_si_001.pdf [file nn2c08435_si_001.pdf]

Supporting Information to: From molecular to multi-asperity contacts: how roughness bridges the friction scale gap

Lucas Frérot,[*,†,‡,§] Alexia Crespo,[¶] Jaafar A. El-Awady,[‡] Mark O. Robbins,[†] Juliette Cayer-Barrioz,[*,¶] and Denis Mazuyer[¶]

†*Department of Physics and Astronomy, Johns Hopkins University, 3400 N Charles Street, Baltimore, Maryland 21218, USA*

‡*Department of Mechanical Engineering, Johns Hopkins University, 3400 N Charles Street, Baltimore, Maryland 21218, USA*

¶*Laboratoire de Tribologie et Dynamique des Systèmes, École Centrale de Lyon, CNRS UMR5513, 69134, Ecully, France*

§*Current Affiliation: Institut for Mikrosystemtechnik, Albert-Ludwigs-Universität, 79110 Freiburg, Germany*

E-mail: lucas.frerot@imtek.uni-freiburg.de; juliette.cayer-barrioz@ec-lyon.fr

Codes and data used to generate figures are available on Zenodo[1].

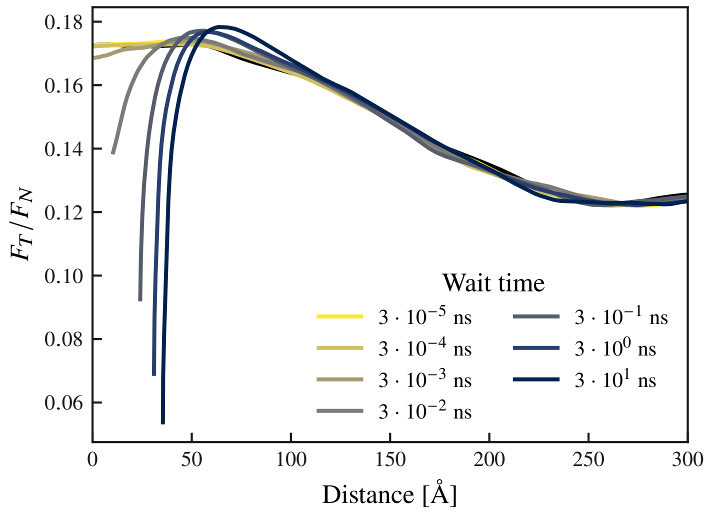

Figure S1: **Friction overshoot peaks in a rough-on-rough system (MD).** Due to the roughness present on both surfaces, the steady-state friction in not constant, but varies as the contact interface changes in the sliding process. Wait time shown are in unscaled units.

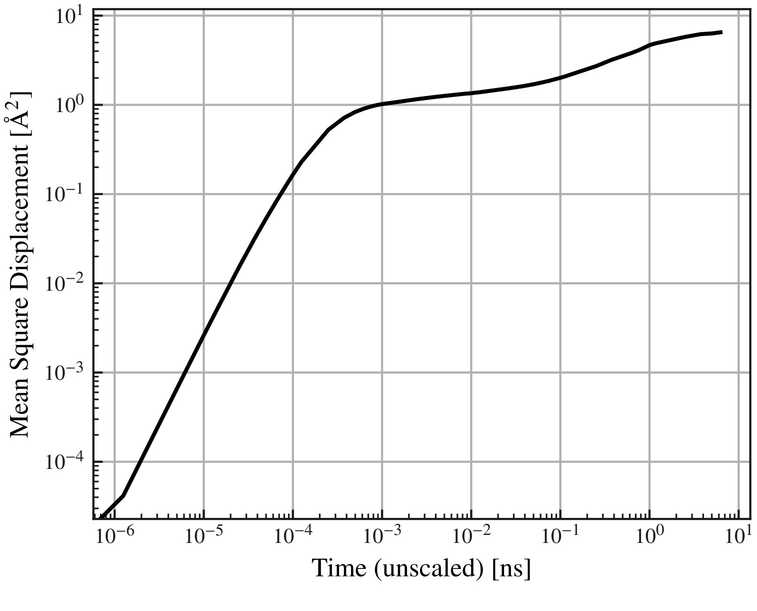

Figure S2: **Mean square displacement of beads (MD), with respect to unscaled time.** The time scaling factor, α for the coarse-grained model used[2] is 7.63. The first characteristic timescale corresponds to the end of the ballistic regime ($t/\alpha \approx 10^{-3}$ ns) and gives the monomer relaxation time. The second characteristic timescale corresponds to the chain relaxation timescale, at $t/\alpha \approx 10^{-1}$ ns $\Rightarrow \tau_{\mathrm{MD}} = 0.8$ ns. The ratio of these characteristic times is the parameter m of our friction model.

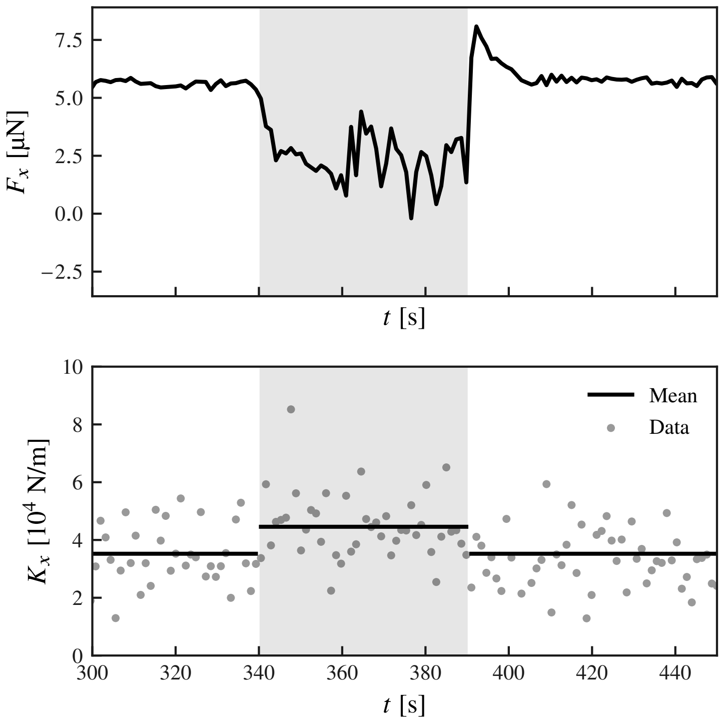

Figure S3: **Structural aging of the interface (experimental).** Simultaneous evolution of the friction force (top) and the tangential stiffness (bottom) during an SHS process. A slight increase in the tangential stiffness, K_x, is measured during the holding stage, which spans 50 s. No variation of the film thickness is detected. [3]

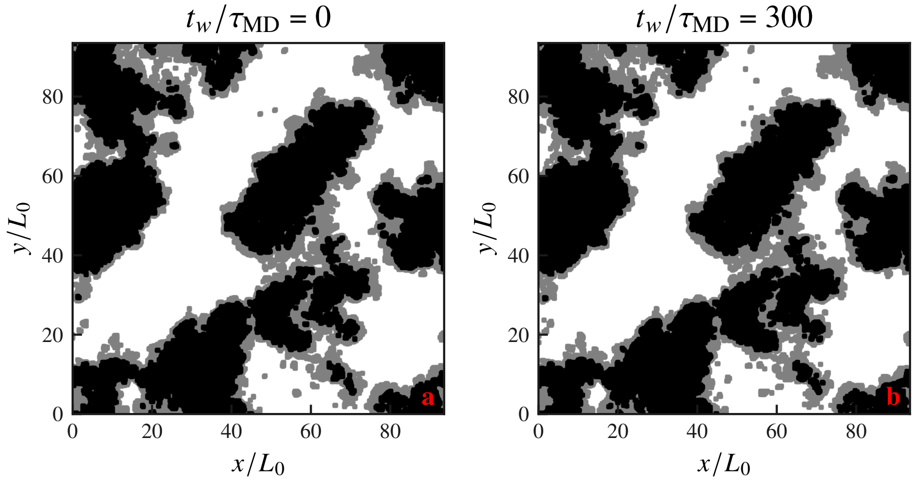

Figure S4: **Contact area evolution during the holding stage (MD).** Black zones indicate repulsive interaction, while gray zones indicate attractive interaction. **a** shows the state of the contact area before the holding stage (at the end of the sliding stage); while figure **b** shows the end of the holding stage for $t_w/\tau_{\mathrm{MD}} = 300$. No significant difference can be observed.

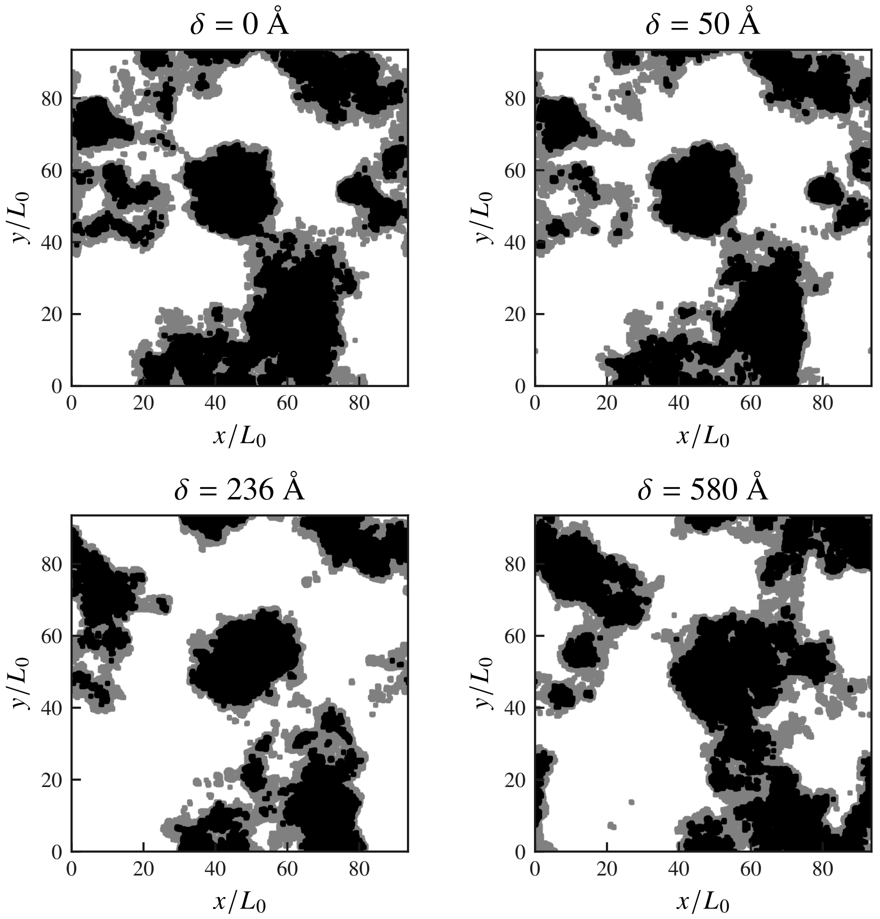

Figure S5: **Contact area evolution in the second sliding stage of the rough-on-rough system (MD).** Each image corresponds to a different value of the sliding distance, δ. On distances of the order of the characteristic scale $D_0 = 3.5\,\mathrm{nm}$, the true contact area is not significantly modified by the shifting of the two rough surfaces.

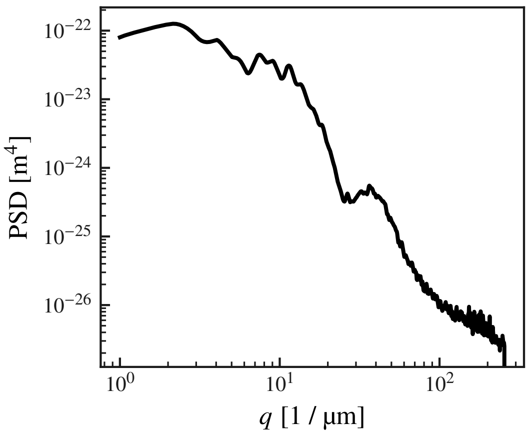

Figure S6: **Power-spectrum density of tribometer surface topography.** Topography was measured with AFM over an area of $1\,\mu\mathrm{m}\times1\,\mu\mathrm{m}$.

References

1. Frérot, L.; Crespo, A.; El-Awady, J. A.; Robbins, M. O.; Cayer-Barrioz, J.; Mazuyer, D. Supplementary Codes and Data to "From Molecular to Multi-Asperity Contacts: How Roughness Bridges the Friction Scale Gap". https://zenodo.org/record/6966730 (accessed 2022-08-05), 10.5281/zenodo.6966730, 2022.

2. Salerno, K. M.; Agrawal, A.; Perahia, D.; Grest, G. S. Resolving Dynamic Properties of Polymers through Coarse-Grained Computational Studies. *Physical Review Letters* **2016**, *116*, 058302.

3. Crespo, A. Compréhension de la tribologie de films limites : De l'organisation moléculaire à la réponse en friction. Ph.D. thesis, École Centrale de Lyon, 36 avenue Guy de Collongue, 69134 Écully, France, 2017.
